# Neuroprotective and anticonvulsant effect of trimetazidine in a PTZ-kindling model of mice through modulation of the IL-1β/IL-1R1 and HMGB-1/TLR-4 axis

**DOI:** 10.3389/fphar.2025.1621729

**Published:** 2025-08-15

**Authors:** Shahnawaz Ahmad, Mohammed Samim, Seema Jain, Divya Vohora, Nidhi

**Affiliations:** ^1^ Department of Pharmacology, School of Pharmaceutical Education and Research, Jamia Hamdard, New Delhi, India; ^2^ Department of Chemistry, School of Chemical and Life Sciences, Jamia Hamdard, New Delhi, India; ^3^ Department of Pharmacology, University College of Medical Sciences, University of Delhi, New Delhi, India; ^4^ Department of Translational and Clinical Research, School of Chemical and Life Sciences, Jamia Hamdard, New Delhi, India

**Keywords:** epilepsy, cognition, trimetazidine, PTZ-kindling, neuroinflammation, oxidative stress, neurotransmitters

## Abstract

**Background:**

Epilepsy is a chronic and complex brain disorder characterized by frequent seizures, cognitive impairments, neuroinflammation, oxidative stress, and imbalances in neurotransmitters. Developing an effective therapeutic intervention to target these pathological interventions remains a challenge. Trimetazidine (TMZ), the most commonly known anti-ischemic agent, has emerged as a promising candidate for its role in epilepsy due to its diverse mechanisms of action. This study investigates the neuroprotective, anticonvulsant, anti-inflammatory, antioxidant, and neuromodulatory effects of TMZ in managing epilepsy.

**Methods:**

Kindling was induced by administering Pentylenetetrazole (30 mg/kg, *i.p*) to Swiss albino mice on every alternate day; TMZ (5, 10, and 20 mg/k *p.o*) or sodium valproate (200 mg/kg *p.o*) was given for 5 weeks. Seizure severity was assessed on the Racine scale, and cognitive function and learning were evaluated using the elevated plus maze and the passive avoidance apparatus. Muscle strength was measured using the rotarod test. Neuroinflammatory biomarkers (IL-1β, IL-1R1, IL-6, NF-κB, TNF-α, HMGB-1, TLR-4), oxidative stress markers (MDA, GSH, SOD, catalase), and neurotransmitter (GABA, glutamate, dopamine, serotonin) levels were estimated in the hippocampus and cerebral cortex using commercially available sandwich ELISA kits.

**Results:**

TMZ, primarily at 10 and 20 mg/kg, significantly reduced seizure scores and improved the transfer latency, step-down latency, and motor abilities in the PTZ-kindled animals. It significantly reduced proinflammatory molecules IL-1β, IL-1R1, IL-6, NF-κB, TNF-α, HMGB-1, TLR-4. Additionally, it increased antioxidant enzyme activity (GSH, SOD, catalase) while lowering MDA levels and restoring GABA, dopamine, and serotonin levels, as well as suppressing glutamate levels, comparable to VPA at 200 mg/kg/day *p.o*.

**Conclusion:**

TMZ, at doses of 10 and 20 mg/kg p.o., demonstrated anticonvulsant and antioxidant activity, suppressed kindling progression, and restored neurotransmitter balance. Furthermore, TMZ has shown anti-inflammatory activity against neuroinflammation during epilepsy.

## 1 Introduction

Epilepsy is one of the most chronic and complex neurological disorders, currently affecting more than 50 million individuals of every age, sex, and race worldwide (Kim et al., 2024; Vezzani et al., 2019). Despite the wide availability of clinically effective anti-seizure medications (ASMs), approximately one-third of the patients are still refractory to seizure control, and 70%–80% of patients experience significant adverse drug reactions with existing pharmacotherapies (Löscher et al., 2020; Zhang et al., 2022). This becomes a significant concern, depicting an urgent need for a new molecule that can interfere with pathophysiological mechanisms rather than providing symptomatic relief (Khatoon et al., 2023).

An epileptic seizure directly impacts the structural and functional properties of the brain’s neuronal network; these changes can significantly affect cognitive functions over time, including memory, attention, and executive function. The severity depends on factors such as the type and frequency of seizures (Devinsky et al., 2018). Multiple effects of ASMs, like sedative effects and central nervous system depressants, can further impair attention, memory, and psychomotor speed and contribute to cognitive decline (Trinka et al., 2019). Additionally, social stigma, chronic stress, and day-to-day life discrimination associated with epilepsy, along with anxiety and depression, can further lead to cognitive decline. Thus, an appropriate balance between seizure control and cognitive side effects is a crucial aspect of the management of epilepsy (Hoda et al., 2021; Dahalia et al., 2024). Furthermore, it is well-documented that 30%–50% of individuals with epilepsy experience symptoms of depression at some point in their lives, significantly impacting their quality of life and overall health. Depression associated with epilepsy often remains undiagnosed and inadequately addressed due to overlapping clinical symptoms, the social stigma surrounding mental health, and the misconception that mood disturbances are merely reactions to coping with a chronic illness. Thus, it should become mandatory to assess depression in epilepsy patients (Qin et al., 2022).

Neuroinflammation plays a critical role in the pathophysiology of epilepsy, leading to the development, propagation, and aggravation of seizures. This mechanism involves activating microglia, astrocytes, and parenchymal cells, which begin to release various inflammatory mediators, including the pro-inflammatory cytokines interleukin-1 beta (IL-1β), interleukin-6 (IL-6), and Tumor Necrosis Factor-Alpha (TNF-α), as well as chemokines. These mediators can modify neuronal excitability by influencing neurotransmitter systems, ion channels, and synaptic function (Vezzani et al., 2022; Tyagi et al., 2023). This activity disrupts the blood-brain barrier, enhancing glutamatergic transmission and the release of excitatory neurotransmitters. This misbalances the transmission of neuroinhibitory gamma-aminobutyric acid (GABA) and neuroexcitatory (glutamate) neurotransmitters, contributing to cognitive dysfunction impairment. Consequently, neuronal excitability increases, facilitating the extravasation of peripheral immune cells into the central nervous system and contributing to sustained neuroinflammation and neuronal damage. Chronic neuroinflammation can also result in structural damage to neurons, including gliosis and neuronal loss, which perpetuates the cycle of inflammation and epilepsy (Foiadelli et al., 2023; Khatoon et al., 2023; Li et al., 2023).

High mobility group box 1 protein (HMGB-1) has recently gained significant attention for its role in initiating neuronal hyperexcitability, modulating neuroinflammation, and influencing cognitive function in epilepsy. This nuclear protein is released extracellularly in response to stress, injury, or infection and acts as a pro-inflammatory cytokine by binding to the toll-like receptor (TLR-4). It has also been found to enhance the release of other pro-inflammatory cytokines and to affect synaptic transmission and plasticity. This interaction represents a potential therapeutic target: blocking HMGB-1 or TLR-4 has been shown to reduce seizure susceptibility and severity in preclinical models (Paudel et al., 2020). In a clinical study conducted on paediatric patients with epilepsy aged 4–17 years, elevated serum levels of HMGB-1 were observed, supporting its role in the pathophysiology of the disease (Kamaşak et al., 2020). Additionally, the activation of the HMGB1/TLR4 axis has been demonstrated in surgically resected drug-refractory brain tissue, indicating its crucial role in the development of seizures (Pauletti et al., 2019; Dahalia et al., 2024). However, the exact mechanisms by which the HMGB1/TLR4 axis contributes to epilepsy remain unclear. Exploring these pathways will be crucial for developing strategies to mitigate seizures and the process of epileptogenesis.

Oxidative stress is increasingly recognized as a critical contributor to the pathophysiology of epilepsy. During seizure episodes, the excessive production of reactive oxygen species (ROS) exceeds the brain’s intrinsic antioxidant defense mechanisms, resulting in mitochondrial dysfunction and neuronal injury (Yu et al., 2021). This imbalance is evidenced by elevated levels of lipid peroxidation products such as malondialdehyde (MDA), along with decreased concentrations of key antioxidants, including reduced glutathione (GSH), superoxide dismutase (SOD), and catalase. Prolonged oxidative damage in various brain regions has been linked to long-term neurological sequelae, including structural changes and cognitive deficits commonly seen in neurodegenerative disorder conditions. The resultant oxidative burden not only contributes to neuronal degeneration through lipid peroxidation but also reduces endogenous antioxidants such as GSH, SOD, and catalase (Liu et al., 2017; Dahalia et al., 2024; Mahmoud et al., 2024). Experimental and clinical studies consistently support the role of oxidative stress in both the initiation and progression of epilepsy pathology. Furthermore, evidence suggests that chronic administration of certain ASMs may exacerbate oxidative damage. For instance, valproic acid has been associated with increased lipid peroxidation, while other agents like phenytoin have shown potential antioxidative effects, as indicated by elevated glutathione reductase activity in treated patients. Despite the efficacy of ASMs in controlling seizures, their long-term impact on redox homeostasis and cognitive function remains a subject of ongoing investigation (Xiang et al., 2021).

TMZ, a clinically active and most widely used anti-ischemic agent for treating stable angina, has emerged as a promising candidate for anticonvulsant and antioxidant therapy due to its multifaceted mechanisms of action (Al-Shorbagy et al., 2021). It works by inhibiting fatty acid β-oxidation, shifting the oxygen demands towards glucose oxidation. This metabolic shift is particularly relevant in epilepsy, where oxidative stress, energy deficits, and mitochondrial dysfunction are key contributors to neuronal hyperexcitability and seizure propagation (Mahmoud et al., 2024).

The anti-inflammatory and antioxidant effects of TMZ play a crucial role in modifying seizure-induced neuroinflammation and oxidative neuronal damage. Its ability to modulate these inflammatory pathways, reduce ROS production, and stabilize mitochondrial membranes underscores its neuroprotective potential in seizure disorders (Terrone et al., 2020). Furthermore, TMZ may exert modulatory effects on ion channels, particularly calcium and potassium channels, which also play a role in neuronal excitability and the generation of convulsive behavior. The compound that can restore the ion channel dysregulation and restore its proper function could hold a significant therapeutic promise. Another remarkable aspect of the anticonvulsant potential of TMZ is its favourable side-effect profile as compared to other conventional ASMs. Many ASMs are associated with drug resistance, cognitive dysfunction, sedation, and liver toxicity, limiting their long-term use. An already established safety and efficacy profile in chronic cardiac conditions suggests that TMZ could be a well-tolerated alternative or an adjunct therapy option for the treatment or management of epilepsy. Despite these promising findings, the exact molecular mechanisms underlying the anticonvulsant effects of TMZ have yet to be clarified entirely. We designed this study to evaluate the neuroprotective and anticonvulsant effects of TMZ in a PTZ-kindling model of seizures in mice.

## 2 Materials and methods

### 2.1 Animals

Forty-eight healthy adult Swiss albino mice of both sexes (24 males and 24 females), aged 7–8 weeks and weighing between 25 and 30 g, were housed in pathogen-free, husk-bedded polypropylene cages (measuring 43 cm × 28.6 cm × 15.5 cm) at the Central Animal House Facility of Jamia Hamdard, under standard laboratory conditions for mice (12h light-dark cycle), and provided continuous access to RO water and a standard pellet diet *ad libitum*. The experimental procedures and animal care were conducted per the guidelines established by the Committee for the Control and Supervision of Experiments on Animals (CCSEA) India, as well as the approved protocol by the Institutional Animal Ethics Committee (IAEC) of Jamia Hamdard, New Delhi, India (Reg. No. and Date of Reg: 173/GO/ReBi/S/2000/CPCSEA, 28 January 2000) (Approval number and Date of approval: Sr no. 1580, 25 March 2019). The findings were reported in accordance with the Animal Research Reporting *In Vivo* Experiments (ARRIVE) guidelines.

### 2.2 Drug administration protocol

Trimetazidine (TMZ), sodium valproate (VPA), and pentylenetetrazole (PTZ) were obtained from Sigma-Aldrich, and all reagents used were of high analytical grade. TMZ was suspended in a 1% carboxymethyl cellulose (CMC) solution in ultrapure water and administered daily at oral dosages of 5, 10, and 20 mg/kg. The PTZ solution was prepared by dissolving it in normal saline (0.9% sodium chloride solution) and was delivered intraperitoneally at a dose of 30 mg/kg once every other day, 1 h after the drug administration treatment (Jain et al., 2011). Control animals received vehicles only (1% CMC, normal saline). VPA was delivered orally daily at a dose of 200 mg/kg. All stock solutions were freshly prepared daily before administration at a volume of 10 mL/kg for 35 days.

### 2.3 Experimental design

Animals were randomly divided into six groups, each containing four male and four female mice. Group I, *The negative control group*: 1% carboxymethyl cellulose (CMC) per oral daily with normal saline (NS) intraperitoneally every alternate day; Group II, *The untreated kindling-induced group*: 1% carboxymethyl cellulose (CMC) per oral daily followed by PTZ (30 mg/kg) intraperitoneally every alternate day; Group III, *Standard control group*: Sodium Valproate (200 mg/kg) per oral daily followed by PTZ (30 mg/kg) intraperitoneally every alternate day; Groups IV, *TMZ5 group*: TMZ (5 mg/kg) per oral daily followed by PTZ (30 mg/kg) intraperitoneally every alternate day; Groups V, *TMZ10 group*: TMZ (10 mg/kg) per oral daily followed by PTZ (30 mg/kg) intraperitoneally every alternate day; Groups VI, *TMZ20 group*: TMZ (20 mg/kg) per oral daily followed by PTZ (30 mg/kg) intraperitoneally every alternate day; for 35 days. The sample size was determined using G*Power software. (Version 3.1.9.3 for Windows 10). The effect size was 2.54, alpha was 0.05, and the power was 0.95.

### 2.4 PTZ-induced kindling in mice

Immediately after administering the PTZ, seizure activity was continuously monitored for 30 min. The seizure intensity was noted using the Racine scale as follows: “Stage 0: no response; Stage 1: ear and facial spasms; Stage 2: myoclonic body jerks without upright position; Stage 3: myoclonic jerks, upright position with bilateral forelimb clonus; Stage 4: tonic-clonic seizures; Stage 5: generalized tonic-clonic seizures, loss of postural control” (Racine, 1972). Mice who experienced stage 4 or stage 5 seizures in three consecutive administrations of PTZ were considered completely kindled, and their treatment was discontinued. Seizures that occurred during the kindling procedure were allowed to stop on their own without any pharmacological intervention. Cumulative mean seizure scores were assessed weekly for each group on days 7, 14, 21, 28, and 35 and reported as mean ± SEM (Khatoon et al., 2021; Sehar et al., 2015).

### 2.5 Transfer latency on the elevated plus maze

The memory and spatial learning capabilities of TMZ were assessed using the elevated plus maze apparatus. It consists of a 25 cm elevated four-armed wooden maze consisting of two open areas and two covered arms (dimensions: 16 cm × 5 cm × 15 cm) bisecting and forming a central square shape of 5 cm × 5 cm. Mice were placed individually at the terminus end of an open arm, and the time taken for each mouse to enter one of the closed arms with all four limbs was defined as Transfer latency. The transfer time for the first-day test was termed the acquisition latency, and the time repeated after 24 h was termed retention latency. A transfer latency of 90 s was assigned to the mouse that failed to enter; this mouse was then assisted in exploring the maze for one more minute before being returned to its cage (Siddiqui et al., 2021).

### 2.6 Step-down latency on passive avoidance apparatus

In this test, the long-term cognitive effects of TMZ were assessed using an inverted glass Petri dish placed in the middle of the apparatus grid floor, which serves as an insulated space or a shock-free zone. The mice were placed individually in this zone and received a mild electroshock upon stepping down from this zone with all four legs. The mouse was trained to stay over the plate for 1 minute. After the training session, the time taken by the mouse to step down from the Petri dish to the grid floor without receiving a shock was recorded as the acquisition latency time. The test was repeated after 24 h, and this was termed as the retention latency time. Mice that stayed on the Petri dish (shock-free zone) for a duration of more than 600 s were assigned a cut-off latency of 600 s (Agarwal et al., 2011).

### 2.7 Rotarod test

Through the rotarod test, we evaluated the chronic effects of TMZ on skeletal muscle strength, coordination, and balance in the mice. The setup contained a rotating rod with a 3 cm diameter fixed 25 cm above the base. Individually, mice were placed on a rod that was continuously rotated at a speed of 20 rotations per minute. The latency for the mice to fall from the rotating rod was recorded in seconds. Each mouse underwent three consecutive trials, and the average latency was calculated. To ensure consistent conditions, the apparatus was cleaned in between each trial (Lubrich et al., 2022).

### 2.8 Tissue preparation

Twenty-four hours after the neurobehavioral assessment, the animals were euthanized with high-pressure, concentrated CO_2_ gas. The brain was carefully removed intact, flushed with ice-cold normal saline, and immediately stored at −80°C. The hippocampus and entire cerebral cortical tissues of the experimental mice brain were excised, weighed, and homogenized separately in 0.1 M phosphate buffer solution (pH 7.4) at a ratio of 10% (w/v), then centrifuged for 20 min at 4°C and 10,000 rpm. Cell debris was discarded, and the supernatant was used to quantify pro-inflammatory molecules (HMGB-1, TLR-4, IL-6, IL-1β, IL-1R1, NF-κB, and TNF-α), oxidative stress markers (SOD, catalase, GSH, and MDA), and neurotransmitters (GABA, glutamate, dopamine, and serotonin) using sandwich ELISA kits obtained from Amplicon Biotech, New Delhi, India, following the manufacturer’s guidelines and protocols. The markers and their respective catalog numbers are: IL-6 (E0049Mo), IL-1β (E00192Mo), IL-1R1 (E2665Mo), TNF-α (E0117Mo), NF-κB (E1350Mo), HMGB-1 (E0523Mo), and TLR-4 (E1663Mo). MDA (E0625Mo), GSH (E0179Mo), SOD (E0290Mo), and Catalase (E0076Mo). GABA (E0360Mo), Glutamate (E1716Mo), Dopamine (E0667Mo), and Serotonin (E1448Mo).

### 2.9 Statistical analysis

The data were analyzed using a one-way ANOVA followed by Tukey’s test, and the software GraphPad Prism (version 9.3) was used to conduct the statistical analysis and create the graphical representations. *p* < 0.05 was set to be considered statistically significant. The normality of the data was tested using the Kolmogorov-Smirnov, Shapiro-Wilk tests, as well as a Quantile-Quantile plot. The results are expressed as mean ± SEM. Cumulative mean seizure scores were analyzed using two-way ANOVA.

## 3 Results

### 3.1 Effect of trimetazidine on seizure score in PTZ-kindled mice

**FIGURE 1:**
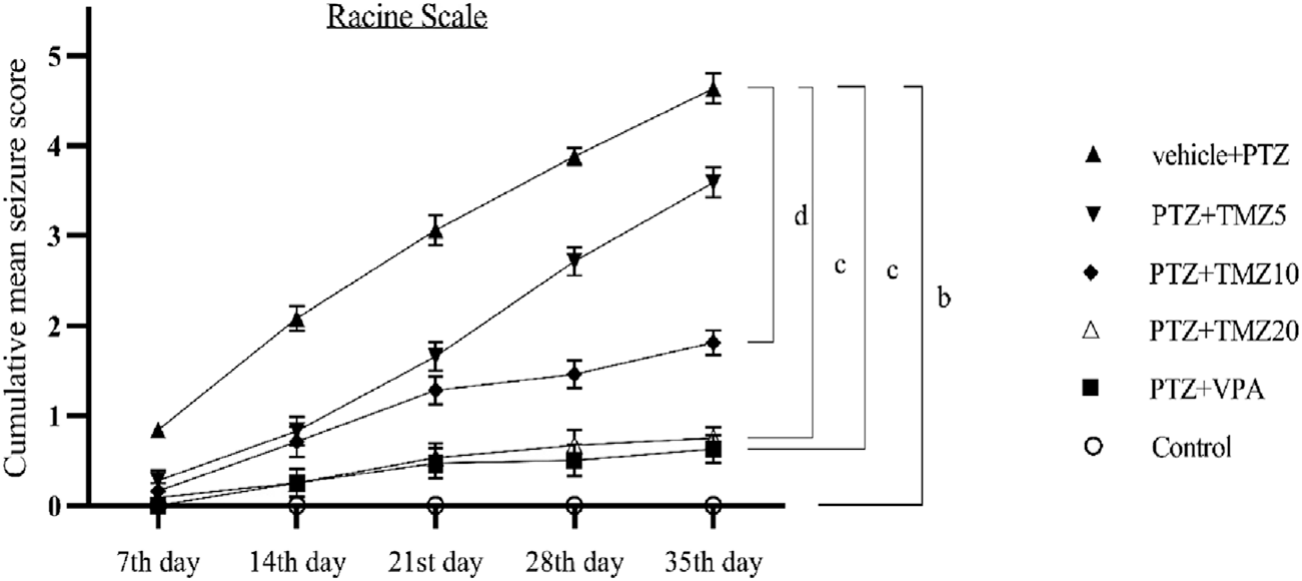
Effect of trimetazidine on pentylenetetrazole-induced kindling. Data were expressed as mean ± SEM (n = 8) and analyzed using a two-way ANOVA followed by Tukey’s Test. ^b^ *p* < 0.001, ^c^ *p* < 0.01, ^d^ *p* < 0.05 when compared to the vehicle + pentylenetetrazole group.

On the last day of the kindling procedure, the anticonvulsant effect of TMZ was assessed using the Racine scale. For each group, cumulative mean seizure scores were calculated and plotted over time. The mean seizure score was significantly higher (*p* < 0.001) in the PTZ-treated group compared to the control group, whereas it was significantly lower (*p* < 0.05 and *p* < 0.01) in the TMZ 10 mg/kg and TMZ 20 mg/kg groups, respectively, compared to the PTZ group (Figure 1).

### 3.2 Effect of trimetazidine on transfer latency

**FIGURE 2:**
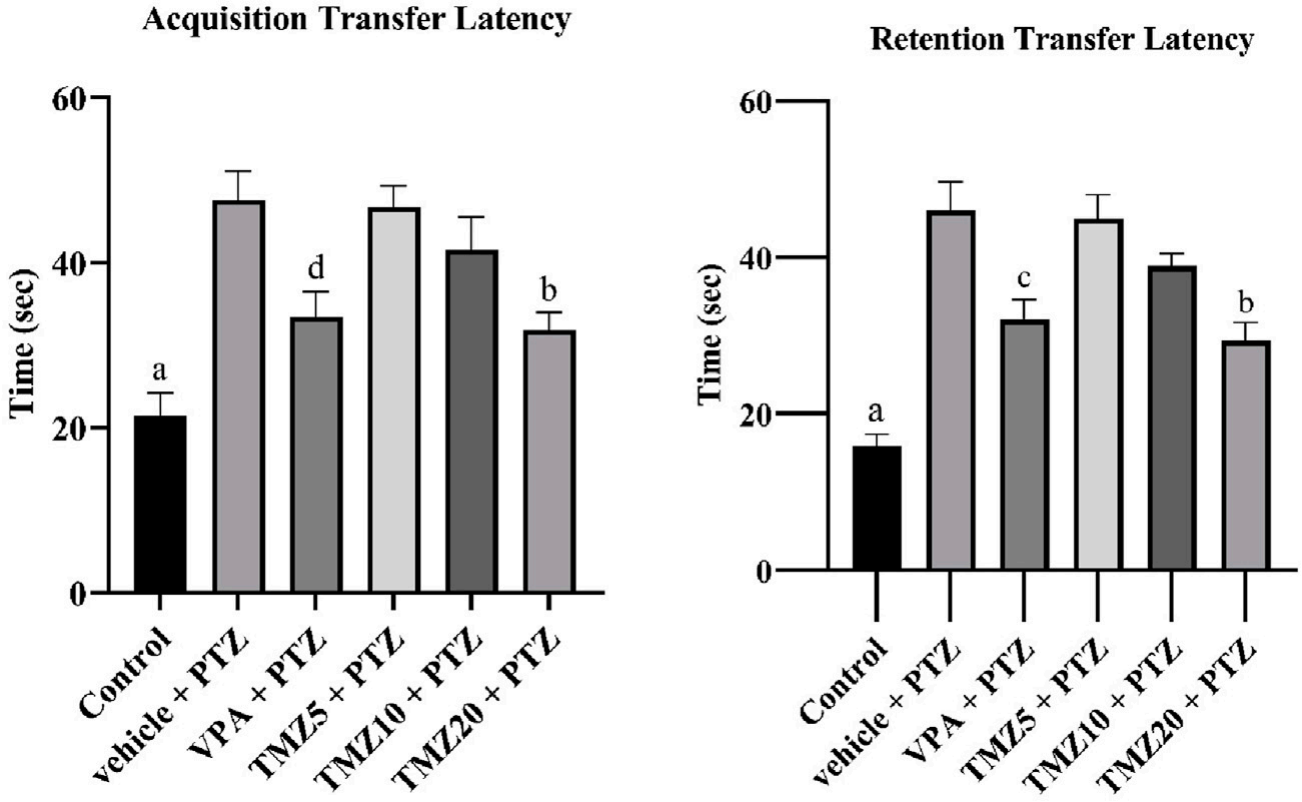
Effects of trimetazidine on transfer latency. Data were expressed as mean ± SEM (n = 8) and analyzed using a one-way ANOVA followed by Tukey’s Test. ^a^ *p* < 0.0001, ^b^ *p* < 0.001, ^c^ *p* < 0.01, ^d^ *p* < 0.05 compared to vehicle + pentylenetetrazole group. A significant decrease in transfer latency time indicates an improvement in memory and learning.

During the transfer latency experiment on the elevated plus maze, the vehicle + PTZ group showed a significantly longer retention latency time (*p* < 0.0001) compared to the control group. The administration of TMZ at 20 mg/kg led to a significant reduction (*p* < 0.01) in acquisition latency and (*p* < 0.001) in retention latency relative to the vehicle + PTZ group (Figure 2).

### 3.3 Effect of trimetazidine on step-down latency

**FIGURE 3:**
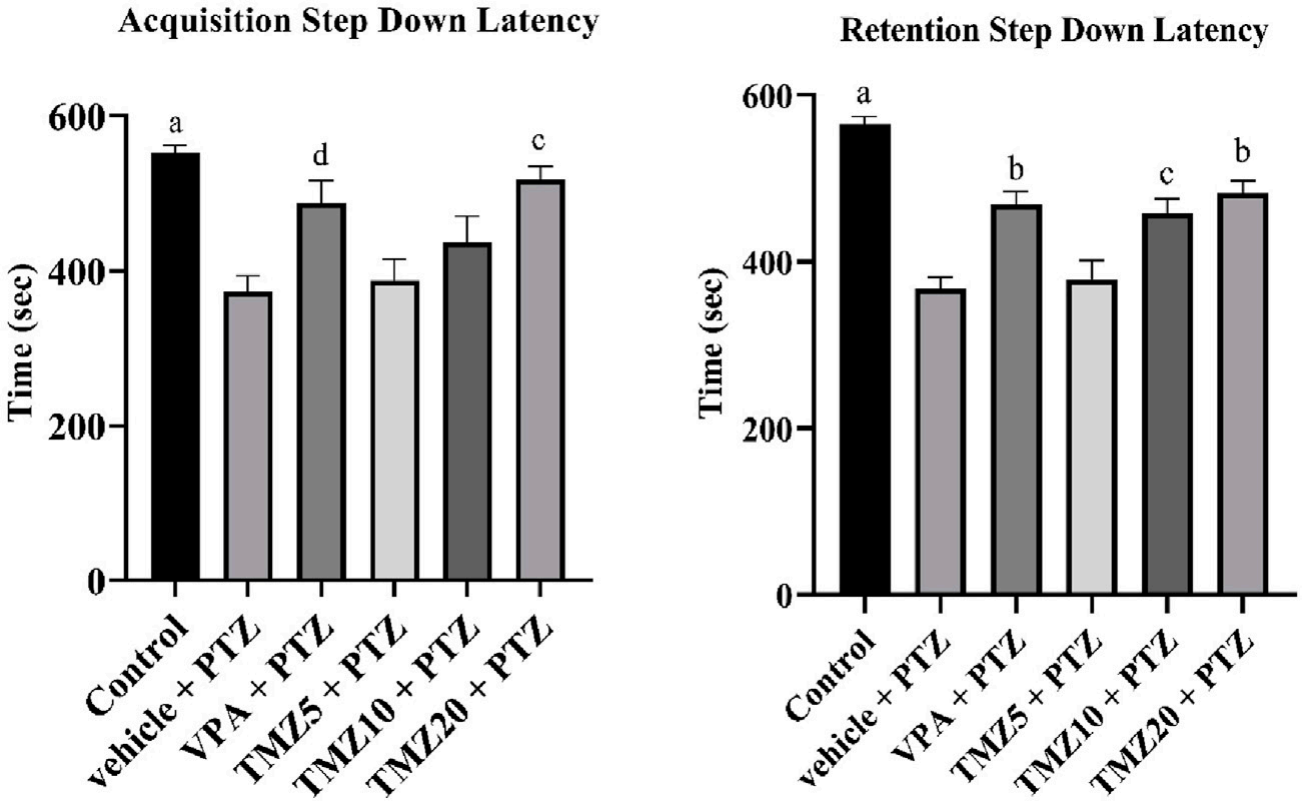
Effects of trimetazidine on step down latency. Data were expressed as mean ± SEM (n = 8) and analyzed using a one-way ANOVA followed by Tukey’s Test. ^a^ *p* < 0.0001, ^b^ *p* < 0.001, ^c^ *p* < 0.01, ^d^ *p* < 0.05 compared to vehicle + pentylenetetrazole group. A significant increase in step down latency time indicates an improvement in memory and learning.

In this passive shock avoidance paradigm, the vehicle + PTZ group exhibited a significantly shorter acquisition and retention latency time (*p* < 0.0001) compared to the control group, indicating cognitive impairment. In the TMZ 20 mg/kg treated group, a significant improvement in memory function was observed (*p* < 0.01), as evidenced by a notable increase in acquisition latency compared to the vehicle + PTZ group. A significant effect was also noted in retention latency for both the TMZ 10 mg/kg (*p* < 0.01) and 20 mg/kg groups (*p* < 0.001) relative to the vehicle + PTZ group (Figure 3).

### 3.4 Effect of trimetazidine on the rotarod test

**FIGURE 4:**
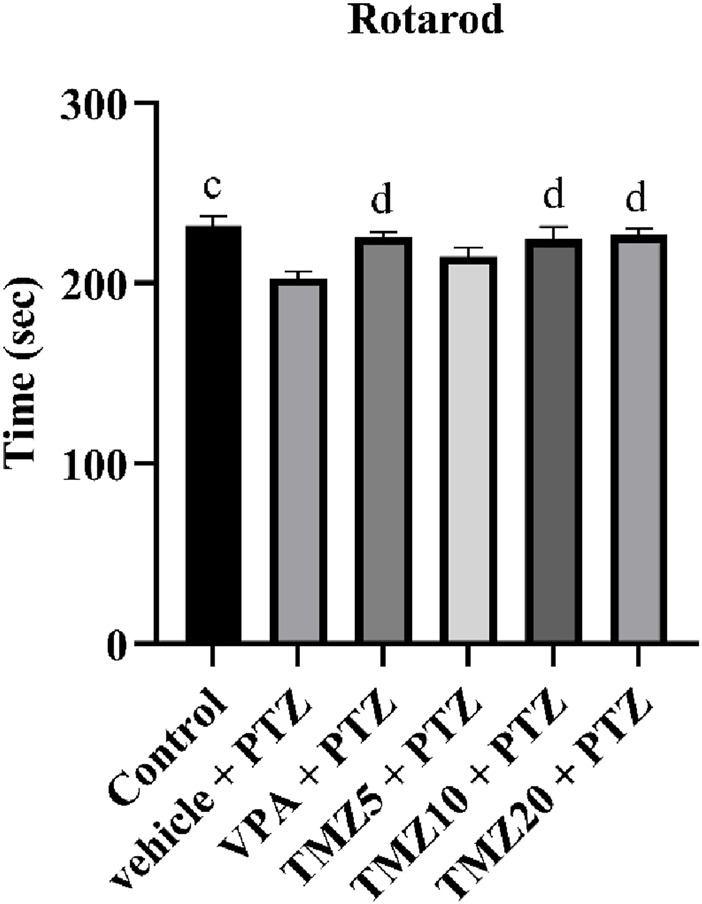
Effects of trimetazidine on rotarod. Data were expressed as mean ± SEM (n = 8) and analyzed using a one-way ANOVA followed by Tukey’s Test. ^c^ *p* < 0.01, ^d^ *p* < 0.05 compared to vehicle + pentylenetetrazole group. A significant increase in latency to fall indicates improved grip strength, motor coordination, and balance.

In this test, the vehicle + PTZ group took a significantly shorter fall latency time (*p* < 0.01) than the control group, suggesting impaired motor coordination and muscle strength (Ali et al., 2004). The TMZ 10 mg/kg and 20 mg/kg treated groups showed a significantly improved fall latency (*p* < 0.05) when compared to the PTZ group (Figure 4).

### 3.5 Effect of trimetazidine on neurotransmitter levels in the hippocampus and cortex of the kindled mice

**FIGURE 5:**
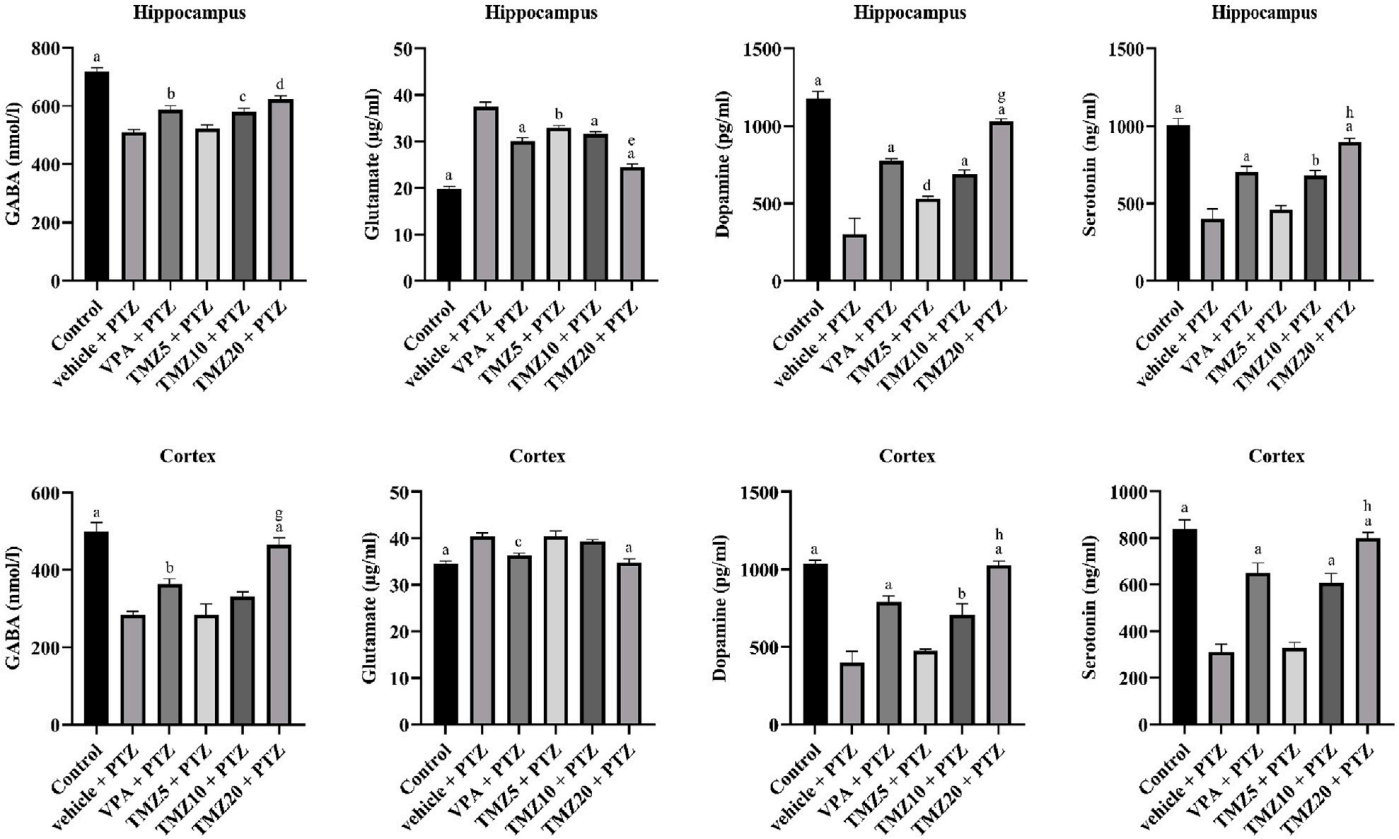
Effect of trimetazidine on neurotransmitters GABA, glutamate, dopamine, and serotonin levels. Data were expressed as mean ± SEM (n = 8) and analyzed using a one-way ANOVA followed by Tukey’s Test. ^a^ *p* < 0.0001, ^b^ *p*<0.001, ^c^ *p* < 0.01, ^d^ *p* < 0.05 compared to vehicle + pentylenetetrazole group; ^e^ *p* < 0.0001, ^g^ *p* < 0.01, ^h^ *p* < 0.05 compared to sodium valproate + pentylenetetrazole group.

The levels of neurotransmitters, including GABA, glutamate, dopamine, and serotonin, were observed in the hippocampus and cortex regions of kindled mice brains. TMZ, administered at doses of 10 mg/kg *p.o*. and 20 mg/kg *p.o*. resulted in a significant increase in hippocampal GABA levels (*p* < 0.01 and *p* < 0.0001, respectively) and a significant rise (*p* < 0.0001) in cortical GABA levels at 20 mg/kg, whereas, a significant decrease in hippocampal glutamate levels (*p* < 0.0001) was observed at both doses of 10 mg/kg and 20 mg/kg, along with a decrease in the cortex at 20 mg/kg when compared to the vehicle + PTZ group. Furthermore, hippocampal dopamine levels were significantly increased (*p* < 0.05) at 5 mg/kg, and (*p* < 0.0001) at 10 mg/kg and 20 mg/kg, as well as in the cortex at 10 mg/kg and 20 mg/kg (*p* < 0.0001), compared to the vehicle + PTZ group. Serotonin levels in the hippocampus were significantly reduced at doses of 10 mg/kg (*p* < 0.001) and 20 mg/kg (*p* < 0.0001), as well as in the cortex (*p* < 0.0001) at both doses, as compared to the vehicle + PTZ group (Figure 5).

### 3.6 Effect of trimetazidine on pro-inflammatory cytokines in the hippocampus and cortex of kindled mice

The pro-inflammatory cytokine level was observed to be significantly higher in the vehicle + PTZ group than in the control group. Administration of TMZ at a dose of 5 mg/kg significantly lowered hippocampal levels of IL-6 (*p* < 0.0001), IL-1β, HMGB-1 (*p* < 0.05), NF-kB (*p* < 0.01), and TLR-4 (*p* < 0.001) when compared to the vehicle + PTZ group. Similarly, administration at a dosage of 10 mg/kg led to a significant decrease in hippocampal IL-6, TLR-4 (*p* < 0.0001), IL-1β, NF-kB (*p* < 0.01), TNF-α, and HMGB-1 (*p* < 0.05) compared to the vehicle + PTZ group. In the hippocampus of the TMZ 20 mg/kg group, significantly lower levels of IL-6, IL-1β, IL-1R1, TNF-α, NF-kB, HMGB-1, and TLR-4 (*p* < 0.0001) were found compared to the vehicle + PTZ group. In the cortex, administration of TMZ at 5 mg/kg significantly reduced IL-6 and TNF-α levels (*p* < 0.0001) when compared to the vehicle + PTZ group. Furthermore, the TMZ 10 mg/kg group also showed significantly reduced levels of IL-6 and TNF-α (*p* < 0.0001) as well as IL-1β (*p* < 0.0001) compared to the vehicle + PTZ group. In the cortex, administration of TMZ at 5 mg/kg significantly reduced IL-6 and TNF-α levels (*p* < 0.0001), and the TMZ at 10 mg/kg group demonstrated significantly reduced levels of IL-6 and TNF-α (*p* < 0.0001), as well as IL-1β (*p* < 0.0001), compared to the vehicle + PTZ group. Additionally, the TMZ 20 mg/kg group significantly decreased levels of IL-6, IL-1β, IL-1R1, NF-kB (*p* < 0.0001), TNF-α, TLR-4 (*p* < 0.001), and HMGB-1 (*p* < 0.05) levels in the cortex compared to the vehicle + PTZ group (Tables 1, 2).

**TABLE 1:** Effect of TMZ on IL-6, IL-1β, IL-1R1, TNF-α, NF-κB, HMGB-1, and TLR-4 levels in the hippocampus of the kindled mice.

Group	Dose (mg/kg)	IL-6 (pg/mL)	IL-1β (pg/mL)	IL-1R1 (ng/L)	TNF-α (ng/mL)	NF-κB (pg/mL)	HMBG-1 (ng/mL)	TLR-4 (pg/mL)
Control	10 (mL/kg)	80.56 ± 6.67	1076 ± 130.71	1491.09 ± 37.62	179.48 ± 34.23	12.50 ± 0.35	367.53 ± 7.78	14.45 ± 0.87
vehicle + PTZ	30	402.78 ± 16.97^i^	2242.25 ± 144.02^i^	1909.84 ± 68.86^i^	1019.06 ± 29.96^i^	16.69 ± 0.35^i^	549.29 ± 17.88^i^	26.4 ± 0.90^i^
VPA + PTZ	200	84.26 ± 6.99^a^	1418.50 ± 89.58^a^	1663.75 ± 39.14^d^	525.31 ± 121.45^c^	14.17 ± 0.35^b^	424.12 ±10.71^a^	13.78 ± 1.02^a^
TMZ5 + PTZ	5	123.15 ± 5.23^a^	1791 ± 51.55^d^	1860.63 ± 40.25	724.79 ± 98.23	14.50 ± 0.40^c^	499.95 ± 10.05^c^	21.02 ± 0.95^b^
TMZ10 + PTZ	10	102.78 ± 7.60^a^	1703.50 ± 43^c^	1809.06 ± 31.11	676.35 ± 91.33^d^	14.36 ± 0.42^c^	426.87 ± 8.25^a^	19.55 ± 0.88^a^
TMZ20 + PTZ	20	71.76 ± 8.95^a^	1199.75 ± 53^a^	1512.97 ± 72.65^a^	266.46 ± 67.75^a^	12.75 ± 0.56^a^	416.43 ± 5.09^a^	13.18 ± 0.93^a^

Data were expressed as mean ± SEM (n = 8) and analyzed by one-way ANOVA followed by Tukey's Test. ^i^ *p* < 0.0001 compared to control group; ^a^ *p* < 0.0001, ^b^ *p* < 0.001, ^c^ *p* < 0.01, ^d^ *p* < 0.05 compared to vehicle + pentylenetetrazole group.

**TABLE 2:** Effect of TMZ on IL-6, IL-1β, IL-1R1, TNF-α, NF-κB, HMGB-1, and TLR-4 levels in the cortex of the kindled mice.

Group	Dose (mg/kg)	IL-6 (pg/mL)	IL-1β (pg/mL)	IL-1R1 (ng/L)	TNF-α (ng/mL)	NF-κB (pg/mL)	HMBG-1 (ng/mL)	TLR-4 (pg/mL)
Control	10 (mL/kg)	37.96 ± 6.13	1728.50 ± 32.99	862.19 ± 121.47	401.35 ± 30.50	8.32 ± 0.18	337.31 ± 9.66	16.08 ± 0.69
vehicle + PTZ	30	234.26 ± 11.15^i^	2483.50 ± 36.19^i^	1655.94 ± 46.57^i^	1203.96 ± 44.94^i^	12.97 ± 0.37^i^	496.1 ± 7.93^i^	27.82 ± 02^i^
VPA + PTZ	200	62.04 ± 7.50^a^	1901 ± 20.09^a^	1031.72 ± 59.71^a^	565.99 ± 52.86^a^	10.57 ± 0.42^c^	430.71 ± 16.93^d^	16.79 ± 1.82^a^
TMZ5 + PTZ	5	111.11 ± 9.87^a^	2268.50 ± 136.38	1606.72 ± 64.89	844.58 ± 45.47^a^	12.21 ± 0.64	485.11 ± 14.33	22.76 ± 1.03
TMZ10 + PTZ	10	79.63 ± 8.88^a^	2184.75 ± 53.22^d^	1363.75 ± 38.98	599.27 ± 43.20^a^	11.32 ± 0.29	479.62 ± 8.57	20.94 ± 1.63
TMZ20 + PTZ	20	52.31 ± 7.95^a^	1781 ± 47.58^a^	991.09 ± 59.29^a^	421.15 ± 23.85^b^	9.39 ± 0.49^a^	425.22 ± 10.49^c^	16.47 ± 0.96^b^

Data were expressed as mean ± SEM (n = 8) and analyzed by one-way ANOVA followed by Tukey's Test. ^i^ *p* < 0.0001 compared to control group; ^a^ *p* < 0.0001, ^b^ *p* < 0.001, ^c^ *p* < 0.01, ^d^ *p* < 0.05 compared to vehicle + pentylenetetrazole group.

### 3.7 Effect of trimetazidine on MDA level in the hippocampus and cortex of kindled mice

The MDA level was significantly raised (*p* < 0.0001) in the vehicle + PTZ group as compared to the control group in both the hippocampus and cortex, indicating an increased level of oxidative stress. TMZ administration at a dose of 10 mg/kg significantly reduced the MDA level in the cortex; however, at a dose of 20 mg/kg, it significantly reduced (*p* < 0.0001) both the hippocampal and cortical levels of MDA as compared to the vehicle + PTZ group (Tables 3, 4).

**TABLE 3:** Effect of TMZ on MDA, GSH, SOD, and catalase levels in the hippocampus of the kindled mice

Group	Dose (mg/kg)	MDA (µmol/L)	GSH (ng/mL)	SOD (ng/mL)	Catalase (ng/mL)
Control	10 (mL/kg)	127.70 ± 3.34	212.95 ± 21.65	6.72 ± 0.15	30.49 ± 1.05
vehicle + PTZ	30	175.34 ± 8.88^i^	132 ± 11.86^i^	4.39 ± 0.32^i^	17.89 ± 0.85^i^
VPA + PTZ	200	144.26 ± 3.37^b^	168 ± 3.11	5.76 ± 0.14^b^	24.17 ± 1.12^c^
TMZ5 + PTZ	5	170.27 ± 2.23	148.27 ± 4.82	4.47 ± 0.15	18.16 ± 1.17
TMZ10 + PTZ	10	156.42 ± 2.55	189.20 ± 4.94^c^	5.08 ± 0.24	22.22 ±1.04
TMZ20 + PTZ	20	129.73 ± 4.76^a^	190.74 ± 4.60^c^	6.43 ±0.21^a^	25.80 ± 1.46^b^

Data expressed as mean ± SEM (n=8). Analyzed by one-way ANOVA followed by Tukey's Test. ^i^ *p* < 0.0001 compared to control group; ^a^ *p* < 0.0001, ^b^ *p* < 0.001, ^c^ *p* <0.01 compared to vehicle + pentylenetetrazole group.

**TABLE 4:** Effect of TMZ on MDA, GSH, SOD, and catalase levels in the cortex of the kindled mice.

Group	Dose (mg/kg)	MDA (µmol/L)	GSH (ng/mL)	SOD (ng/mL)	Catalase (ng/mL)
Control	10 (mL/kg)	76.35 ± 9.30	168.67 ± 5.07	7.53 ± 0.22	25.9 ± 1.04
vehicle + PTZ	30	145.95 ± 5.73^i^	82.76 ± 7.88^i^	5.96 ± 0.21^i^	12.98 ± 1.07^i^
VPA + PTZ	200	111.15 ± 5.45^c^	118.15 ± 5.37^d^	7.04 ± 0.13^b^	18.39 ± 0.99^d^
TMZ5 + PTZ	5	123.99 ± 3.54	99.62 ± 8.40	6.19 ± 0.14	11.35 ± 1.94
TMZ10 + PTZ	10	114.19 ± 5.10^c^	126.58 ± 7.81^b^	6.67 ± 0.12^d^	14.28 ± 10
TMZ20 + PTZ	20	80.07 ± 4.86^a,g^	163.40 ± 7.02^a,f^	7.38 ± 0.14^a^	18.81 ± 1.14^d^

Data expressed as mean ± SEM (n = 8). Analyzed by one-way ANOVA followed by Tukey's Test. ^i^ *p* < 0.0001 compared to control group; ^a^ *p* < 0.0001, ^b^ *p* < 0.001, ^c^ *p* < 0.01, ^d^ *p* < 0.05 compared to vehicle + pentylenetetrazole group; ^f^ *p* < 0.001, ^g^ *p* < 0.01 compared to pentylenetetrazole + sodium valproate group.

### 3.8 Effect of trimetazidine on GSH, SOD, and catalase levels in the hippocampus and cortex of kindled mice

The levels of GSH, SOD, and catalase were significantly lower (*p* < 0.0001) in the vehicle + PTZ group in comparison to the control group in both the hippocampus and cortex, demonstrating reduced antioxidant activity. Hippocampal GSH levels were significantly higher (*p* < 0.01) in the TMZ 10 mg/kg and 20 mg/kg groups; SOD levels were elevated (*p* < 0.0001) in the TMZ 20 mg/kg; catalase levels increased (*p* < 0.001) in TMZ 20 mg/kg compared to the vehicle + PTZ group. In the cortex, GSH levels were elevated significantly at TMZ 10 mg/kg (*p* < 0.001) and TMZ 20 mg/kg (*p* < 0.0001). SOD levels were also increased significantly (*p* < 0.05 and *p* < 0.0001) at the same doses. Additionally, catalase levels were significantly higher (*p* < 0.05) at 20 mg/kg when compared to the vehicle + PTZ group (Tables 3, 4).

## 4 Discussion

The current study investigated the anticonvulsant, neuroprotective, antioxidant, anti-inflammatory, and neuromodulatory effects of TMZ in PTZ-induced kindled mice, a well-established experimental model of chronic seizures used for evaluating ASMs (Alachkar et al., 2020). The results revealed the therapeutic potential of TMZ, primarily at 10 and 20 mg/kg doses, in reducing seizure severity, protecting cognitive and motor dysfunctions, reducing oxidative stress, controlling brain inflammation, and balancing neurotransmitter homeostasis in the hippocampus and cortex. TMZ modulates cellular energy metabolism, prevents mitochondrial dysfunction, and maintains ATP synthesis under hypoxic conditions by enhancing glucose utilization and inhibiting fatty acid oxidation, reducing neuronal hyperexcitability, and stabilizing voltage-gated sodium and calcium channels (Kamranian et al., 2023; Khan et al., 2024; Nadeem et al., 2025). These effects prevent excessive neuronal firing and align with previous findings on the neuroprotective effects of TMZ in seizure models (Jain et al., 2011). TMZ has shown anticonvulsant activity in pilocarpine and PTZ-induced seizure models, improving glutamate homeostasis (Kamisah and Che Hassan, 2024).

TMZ, mainly at 10 and 20 mg/kg, significantly improved acquisition and retention latency time in the elevated plus maze and improved step-down latency time on the passive avoidance tests apparatus, enhancing spatial memory similar to the VPA + PTZ-treated group. This cognitive effect of TMZ comes from its antioxidant and anti-inflammatory properties, which protect the brain against PTZ-induced neurodegeneration and support synaptic plasticity and memory retention (Al-Shorbagy et al., 2021). Additionally, TMZ modulates mitochondrial redox function, maintains glutamate homeostasis (Kolik et al., 2017; Mohamed et al., 2020), and supports memory-related signaling pathways, showing protective effects in epileptic rodent models (Mohamed et al., 2020; Al-Shorbagy et al., 2021).

In the rotarod test, the TMZ 10 mg/kg and TMZ 20 mg/kg treated groups showed significant improvements in motor coordination and balance as compared to the PTZ group. It is likely due to the neuroprotective effect of TMZ towards oxidative damage and neuroinflammation. However, previous studies reported that TMZ combined with valproate impaired motor coordination in mice, suggesting a pharmacodynamic interaction rather than a direct motor effect (Borowicz-Reutt and Banach, 2022). Previous literature also suggests that TMZ alone and in combination with valproate can cause drug-induced reversible Parkinsonism, especially in the elderly, and patients with neurodegenerative disease (Brugger et al., 2016; Dy et al., 2020). Conversely, TMZ demonstrated neuroprotective effects in ischemic spinal cord injury models, improving motor function recovery (Baltalarli et al., 2000). Additionally, TMZ has demonstrated protective effects under hypoxic and hyperthermic conditions, reducing stress markers and improving endurance (Vezzani et al., 2019).

Elevated MDA levels with reduced GSH, SOD, and catalase levels in the vehicle + PTZ group indicated higher oxidative stress in the hippocampus and cortex. TMZ significantly reduced MDA levels and elevated GSH, SOD, and catalase levels at the dose of 20 mg/kg, showing the most prominent effects. TMZ exerts antioxidant effects by scavenging free radicals, enhancing mitochondrial metabolic function, and preventing lipid peroxidation. In animal models of myocardial ischemia-reperfusion injury, TMZ treatment lowered MDA levels and enhanced GSH concentrations in mitochondrial tissues, protecting against oxidative damage (Meng and Yao, 2020; Al-Shorbagy et al., 2021).

There is also a close relationship between female sex steroid hormones and epilepsy. These hormones have been shown to impact neuronal excitability, potentially complicating oxidative stress and neurotransmitter outcomes. Estrogen tends to be proconvulsant, while progesterone and its metabolites are anticonvulsant, mainly influencing N-methyl-D-aspartate (NMDA) and non-NMDA types of glutamate receptors (Taubøll et al., 2015).

Increased concentrations of HMGB-1, TLR-4, IL-6, IL-1β, IL-1R1, NF-κB, and TNF-α in the vehicle + PTZ group indicate significant neuroinflammation in the hippocampus and cortex. TMZ (20 mg/kg) markedly reduced these pro-inflammatory cytokines and signaling molecules, validating prior research that demonstrated that TMZ inhibits IL-6, IL-1β, and TNF-α production in the hippocampal tissues of epileptic rats (Abouelleil et al., 2022). While there is no direct evidence addressing the effect of TMZ on IL-1R1, it likely diminishes IL-1R1 activation by inhibiting IL-1β signaling pathways (Mohamed et al., 2020). TMZ also suppresses TLR-4/NF-κB signaling, reducing pro-inflammatory cytokine release and inhibiting the HMGB-1/TLR-4 axis, a key pathway in neuroinflammation. By blocking NF-κB activation, TMZ prevents the transcription of inflammatory genes, protecting neurons from inflammation-induced apoptosis (Al-Shorbagy et al., 2021; Majid et al., 2025). TMZ further reduces TLR-4 activation and downstream NF-κB signaling. It may also prevent the release of HMGB-1 from neurons and glial cells, thus lowering neuroinflammation and neuronal damage (Mohamed et al., 2020; Vezzani et al., 2016).

A recent subset of monocytes, CD14^−^ and CD16^−^ monocytes, characterized by the absence of their surface markers CD14 and CD16, is gaining increased attention for their role in neuroinflammatory states associated with chronic conditions such as epilepsy and neurodegenerative diseases. Upon infiltration of the Blood Brain Barrier (BBB), these monocytes get differentiated and release inflammatory cytokines and ROS that exacerbate glycolysis and neuronal hyperexcitability, stimulating pro-inflammatory functions. Understanding this complex interplay between immune function and cellular metabolism may provide novel targets for therapeutic intervention in seizure disorders and chronic neuroinflammation (Ruiz et al., 2025).

VPA, a broad-spectrum antiepileptic drug used in this study as a standard comparator, reduced oxidative damage by significantly lowering MDA levels while enhancing GSH, SOD, and catalase. It restored the neurotransmitter levels of GABA and Glutamate, which are essential for seizure suppression and behavioural stabilization, and downregulated inflammatory mediators by decreasing the expression of pro-inflammatory cytokines such as IL-6, IL-1β, IL-1R1, TNF-α, HMGB1, TLR4, and NF-κB signaling that contribute to neuronal hyperexcitability and blood-brain barrier disruption, thereby strengthening its multifaceted neuroprotective role (Rosenberg, 2007; Ximenes et al., 2013; Ahlatcı et al., 2022).

The PTZ-induced kindling resulted in significantly decreased levels of GABA, dopamine, and serotonin, along with increased levels of glutamate in both the hippocampus and cortex of mice. TMZ at 20 mg/kg successfully restored GABA, dopamine, and serotonin levels while reducing glutamate levels, consistent with prior research indicating that TMZ lowered hippocampal glutamate levels during pilocarpine-induced seizures, thus preventing excitotoxicity and neuronal injury. In ischaemic retinal models, TMZ reduced extracellular glutamate by promoting glutamate reuptake via glial transporters (Payet et al., 2004). The balance between excitatory (glutamate) and inhibitory (GABA) neurotransmission is crucial for neuronal homeostasis. TMZ influences this equilibrium by modulating the activity of GABA transaminase (GABA-T) and glutamate decarboxylase (GAD), which enhances GABA synthesis and reduces glutamate excitotoxicity (Kamisah and Che Hassan, 2024; Borowicz-Reutt and Banach, 2022). Additionally, the restoration of dopamine levels highlights the development of mesolimbic and nigrostriatal pathways, which are usually disturbed in seizure disorders (Liu et al., 2018). While modifying serotonin transporter (SERT) levels differently across various diseases, TMZ also raised serotonin (5-HT) levels in serum and platelets in models of myocardial infarction and depression (Liu et al., 2017). The multi-faceted ability of TMZ to modulate oxidative stress pathways, energy metabolism, neuroinflammatory signaling, and neurotransmitter levels was observed, further demonstrating its potential neuroprotective therapeutic effects. However, the study has certain limitations, primarily the lack of long-term follow-up to evaluate the sustained efficacy and safety of TMZ. Furthermore, this study was conducted using an animal model, which may not adequately represent the complex pathophysiology of human epilepsy and its associated cognitive deficits and emotional issues. Further research, including clinical trials and a more extensive investigation of molecular pathways, will be essential to validate these findings.

## 5 Conclusion

This study demonstrates that trimetazidine (TMZ) provides significant neuroprotective effects in a PTZ-induced kindling model of chronic epilepsy, evidenced by improved cognitive and motor function and reduced seizure severity. These therapeutic effects are associated with alterations in several proteins involved in neuroinflammation, oxidative stress, and neurotransmission. TMZ treatment significantly reduced levels of pro-inflammatory mediators, including IL-6, IL-1β, IL-1R1, TNF-α, HMGB1, TLR-4, and the transcription factor NF-κB. In parallel, oxidative stress was attenuated, as indicated by decreased malondialdehyde (MDA) levels and elevated antioxidant markers, such as glutathione (GSH), superoxide dismutase (SOD), and catalase. Additionally, TMZ modulated neurotransmitter levels by increasing the levels of GABA, dopamine, and serotonin and decreasing the levels of glutamate. Notably, TMZ at 20 mg/kg demonstrated therapeutic effects comparable to those of sodium valproate at 200 mg/kg, thereby strengthening its potential as a treatment option. Clinical studies are essential to validate these preclinical findings, evaluate long-term safety and potential drug interactions, and determine whether TMZ is suitable as a monotherapy or as an adjunctive therapy for epilepsy. While these findings support TMZ’s therapeutic potential in the management of epilepsy, the use of ELISA-based quantification limits insight into specific signaling mechanisms, receptor interactions, and cellular localization. Future studies employing pathway-specific analyses and advanced molecular techniques are essential to clarify its mechanisms and explore its broader neuroprotective roles in other neurological disorders beyond epilepsy.

## Data Availability

The original contributions presented in the study are included in the article/supplementary material, further inquiries can be directed to the corresponding author.
